# Modulated Autophagy by MicroRNAs in Osteoarthritis Chondrocytes

**DOI:** 10.1155/2019/1484152

**Published:** 2019-05-08

**Authors:** Yinghao Yu, Jijun Zhao

**Affiliations:** Department of Orthopedics, The Affiliated Wuxi People's Hospital of Nanjing Medical University, Wuxi 214000, Jiangsu Province, China

## Abstract

Osteoarthritis (OA) is a chronic joint disease characterized by articular cartilage regression. The etiology of OA is diverse, the exact pathogenesis of which remains unclear. Autophagy is a conserved maintenance mechanism in eukaryotic cells. Dysfunction of chondrocyte autophagy is regarded as a crucial pathogenesis of cartilage degradation in OA. MircoRNAs (miRNAs) are a category of small noncoding RNAs, acting as posttranscriptional modulators that regulate biological processes and cell signaling pathways via target genes. A series of miRNAs are involved in the progression of chondrocyte autophagy and are connected with numerous factors and pathways. This article focuses on the mechanisms of chondrocyte autophagy in OA and reviews the role of miRNA in their modulation. Potentially relevant miRNAs are also discussed in order to provide new directions for future research and improve our understanding of the autophagic network of miRNAs.

## 1. Introduction

OA is a common chronic joint disease, reducing the function of joints in middle-aged and elderly individuals [1]. As society ages, the prevalence of OA has increased globally. The etiological factors of OA are diverse, including the female sex, aging, obesity, joint injury, mechanical pressure, heredity, etc. [2–4]. However, the precise pathogenesis of OA remains unclear. Subchondral osteosclerosis and synovitis are considered contributors to the progression of OA. Additionally, due to characteristic and pathological changes in the wear and degeneration of articular cartilage, it has been widely recognized as the principal tissue involved in OA [5]. Autophagy, a highly conserved maintenance mechanism, is vital for endochondral homeostasis and cell survival [6]. It executes a strict quality control function by degrading damaged or dysfunctional organelles or macromolecules and recycling the products. Aberrant expression of autophagy-related genes (ATGs) and dysfunction of autophagy are observed during OA progression [7, 8]. Inhibition of autophagy is considered to be associated with OA-related cartilage degeneration and chondrocyte apoptosis. In consideration of its protective and antiapoptotic functions [9, 10], chondrocyte autophagy has gradually become a hotspot in OA research.

miRNAs are endogenous, noncoding, and single-stranded RNAs, comprising approximately 22 nucleotides. As important modulators of gene expression, miRNAs mediate the posttranscriptional regulation of protein-coding genes in biological processes by binding to the 3′-untranslated region (3′-UTR) of specific targeted mRNAs. Many miRNAs have been analyzed to explore their functions and mechanisms in OA [11–13]. Through in-depth research, a growing number of miRNAs have been confirmed to regulate autophagy in OA chondrocytes. Since cartilage loss is irreversible, it is particularly important to investigate the undiscovered mechanisms of miRNAs in the maintenance of steady autophagy.

Focusing on mechanisms of chondrocyte autophagy in OA, this review aims to summarize the recent advances of miRNAs which have been confirmed to be involved in the regulation of autophagy. In addition, we discuss a series of miRNAs whose involvement is uncertain and which have aberrant expression with specific targets, in order to bring attention to the exploration of potential mechanisms and improve the understanding of the regulatory network of miRNAs in autophagy.

## 2. Chondrocyte Autophagy

### 2.1. General Processes

**Figure 1:**
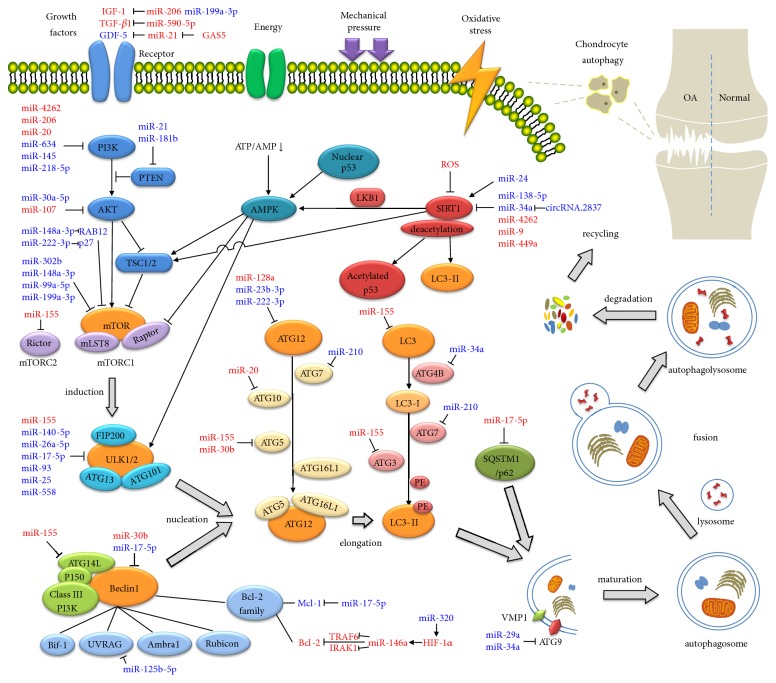
miRNAs, specific targets and signaling pathways in the process of chondrocyte autophagy in OA. Note: miRNAs highlighted in red represent confirmed mechanisms while those highlighted in blue represent hypothesized mechanisms.

Autophagy is a dynamic and sequential process in eukaryotic cells that principally involves the following events: induction, nucleation, elongation, maturation, fusion, and degradation. ATGs, in addition to coded proteins, mediate the entire process (Figure 1). In mammalian cells, autophagy begins with the formation of an uncoordinated 51-like kinase (ULK) complex, which consists of ULK1/2, ATG13, ATG101, focal adhesion kinase (FAK), and family interacting protein of 200 kDa (FIP200). The complex is recognized as a target of mammalian target of rapamycin (mTOR). Under certain circumstances the combined mTOR and ULK1/2 proteins dissociate, resulting in complex activation and phagophore initiation [14]. The class III PtdIns3K (PI3K) complex, composed of class III PI3K, Beclin1, ATG14L, p150, and a series of related modulators, mediates the following nucleation of autophagic vesicles. Because of Beclin1, these related modulators are recruited and activated, including Bax-interacting factor 1 (Bif-1), ultraviolet irradiation resistant-associated gene (UVRAG), activating molecule in Beclin1-regulated autophagy (Ambra1), and Rubicon protein [15]. Additionally, as an essential family of apoptosis-related proteins, the Bcl2 family, which plays a dual role in apoptosis, can also modulate autophagy through Beclin1 [16]. ATG12 and microtubule-associated protein 1 light chain 3 (LC3) can then be recognized as ubiquitin-like protein conjugation systems, which are required during the elongation and maturation of autophagosome [15]. The ATG16L1-ATG12-ATG5 complex is formed through successive conjugations of several ATGs, including ATG12, ATG7, ATG10, ATG5, and ATG16L1. While LC3 is first cleaved into its cytosolic form (LC3-I) by ATG4B [17], LC3-I becomes successively bound to ATG7, ATG3, and phosphatidylethanolamine (PE), creating a lipidated form termed LC3-II. During the process, the ATG12 complex is vital to the LC3 system, indicating that it is required by LC3 to complete subsequent assembly. The specific dependency lies in the targeting of the LC3 lipidation site and formation of an amide bond between LC3-I and PE [18, 19]. Moreover, ATG4B is also identified to maintain the critical function of compensating for defects in lipidation and PE deconjugation processes [17, 20]. The two additional important structures are Atg9 and vacuole membrane protein 1 (VMP1). They are essential transmembrane proteins which directly participate in the elongation and maturation process outside of the complex assembly [15]. These are subsequently assembled together to form a mature autophagosome membrane. Completion of the fusion of the mature autophagosome and lysosome results in formation of the autophagolysosome and degradation of the cell contents.

### 2.2. Signaling Pathways in OA Chondrocytes

A number of essential signaling pathways are involved in the process of autophagy (Figure 1). The class I PI3K-protein kinase B-mTOR (PI3K/AKT/mTOR) pathway is most well-known, acting as a principal regulator in autophagy. mTOR can couple to Raptor or Rictor, correspondingly forming the mTOR complex 1 (mTORC1) or mTOR complex 2 (mTORC2), both of which are able to regulate the process of autophagy [21]. The activated upstream signals from the PI3K/AKT pathway can be integrated by mTORC1 to further suppress autophagy by combining with the ULK complex. Furthermore, AMP-activated protein kinase (AMPK) is a signal site that has been identified and once activated; it directly promotes autophagy and protects chondrocytes from degeneration. Under conditions of energy depletion, which are characterized by a low ATP/AMP ratio, AMPK is able to activate ULK1 [22]. Meanwhile, other signaling pathways of AMPK are also able to downregulate mTORC1 activity through phosphorylation of the Raptor component and an intermediate factor named the tuberous sclerosis complex 1/2 (TSC1/2) [23]. Sirtuin-1 (SIRT1), regarded as a prolongevity factor, mediates progression of both senescence and autophagy. Interventions in autophagy result from direct actions towards a particular endpoint. Cascade reactions of SIRT1 in the modulation of AMPK and mTOR pathways have already been proposed [24, 25]. Furthermore, on account of its deacetylase activity, SIRT1 protects chondrocytes from oxidative stress-mediated death via increased levels of LC3-II [26], also interfering with acetylated p53 and downstream genes, including Bax and Bcl-2 [27, 28]. Interestingly, p53 holds dually positive and negative influences in the induction of autophagy. Activation of p53 in the nucleus induces autophagy by activating AMPK and then inhibiting mTORC1, the inverse of cytoplasmic p53 [29, 30]. Indeed, activation of mTOR and inhibition of autophagy in OA cartilage have already been clarified [31, 32], indicating a crucial pathogenic mechanism. Complex and mutual interfering modes are emerging in the network of autophagy regulation during the progression of OA [33], implicating PI3K/AKT/mTOR, AMPK, SIRT1, P53 pathways and a series of related growth factors, cytokines, and proteins, which are still under intense investigation.

## 3. miRNAs in Autophagy

### 3.1. Biological Synthesis and Functional Mode

Since the discovery of miRNAs, they have been investigated across various fields due to their conserved sequences. The biological synthesis of miRNAs is precisely regulated, beginning with coding genes. In general, coded genes are recognized as being independent. However, in some situations, they may be located in the intronic regions of other genes [34]. Generally, RNA polymerase II mediates the classical transcription of miRNA genes [35]. After formation of a primary RNA (pri-miRNA) in the nucleus, a protein complex consisting of Drosha and DiGeorge syndrome critical region gene 8 (DGCR8) cleaves it into a hairpin of approximately 70 nucleotides [36], which is a precursor of miRNA (pre-miRNA). Exportin-5 then acts as a transport molecule to export pre-miRNA from the nucleus to the cytoplasm [37]. Once completed, the stem-loop structure of the pre-miRNA is further processed by Dicer [38], a type of RNase III, with approximately 22 nucleotides of double-stranded miRNA remaining. The guide strand of mature miRNA stably combines with proteins of the argonaute (AGO) family, forming a biological complex termed miRNA-induced silencing complex (mRISC) [13, 39], while the other passenger strand is rapidly degraded.

The behavior of the mRISC that remains can demonstrate how mature miRNA operates. mRISC is able to recognize certain genes and bind to the 3′-UTR of targeted mRNAs. Interestingly, the degree of complementary of base pairing transpires to be the determinant of the degree of regulation. This explains why mRNA degradation occurs rather than attenuation of protein translation in some circumstances [40]. On account of the pivotal role of the first 2~8 bases of miRNA sequence in target binding [41], partial complementary base pairing triggers inhibition of mRNA translation, whereas perfect complementarity induces cleavage and degradation [13, 42].

Strictly speaking, miRNAs become crucial factors in the pathogenesis of OA due to their widespread target genes. Increasing numbers of miRNAs have been identified as regulating different processes in chondrocyte autophagy through the targeting of particular ATGs, signaling pathways or other related proteins and mediating interventions in endochondral homeostasis (Figure 1).  Table 1 displays a list of miRNAs and their targets that have been identified.

### 3.2. miR-155

Based on an integrated omics analysis, miR-155 was found to be highly upregulated in OA cartilage [68]. Considering previous findings in the induction of autophagy by miR-155 via mTOR signals [69], researchers have already conducted studies to explore the autophagic mechanism in human chondrocytes. They verified that miR-155 did participate in the downregulation of autophagy by targeting several ATGs, including ULK1, FOXO3, ATG3, ATG5, ATG14, GABARAPL1 and MAP1LC3 [43]. miR-155 significantly suppressed both mRNA and protein levels of these ATGs, while silencing miR-155 demonstrated the converse. Simultaneously, the overexpression of miR-155 also decreased the conversion of LC3-I, which is essential for the elongation and enclosure of autophagosomes. Regrettably, D'Adamo et al. [43] predicted the matched targets and other genes but did not conduct further bidirectional verification. What was unexpected was that mTOR activity suppressed by miR-155 seemed to be contradictory to downregulation of autophagy in chondrocytes. Inhibition of mTOR activity was achieved via an important constituent part of mTORC2 named Rictor [70], possibly a target of miR-155 which was able to phosphorylate AKT and activate mTORC1 [44]. Directly targeting regulation of ATGs may explain the differences. Sufficient biological efficiency of miR-155 through downstream target genes independently results in a decrease in the degree of autophagy, without concerning mTOR activity. Actually, further studies are required in order to gain a better understanding of the controversial mechanism of miR-155 in chondrocyte autophagy.

### 3.3. miR-30b

It is of interest that the miR-30 family demonstrates a specific role in autophagy. The family members, including miR-30a, miR-30b, miR-30c, miR-30d, and miR-30e, have been reported to directly bind to the 3′-UTR of Beclin1 and greatly impacts phagophore nucleation [46]. ATG16L1, which participates in elongation and enclosure, is also involved [48]. In particular, of all family members, miR-30b is the most prominent in the regulation of chondrocyte autophagy. Chen et al. [45] constructed differential models of autophagy with tumor necrosis factor-*α* (TNF-*α*), 3-methyladenine and rapamycin in ATDC5 cells, confirming the interaction of Beclin1 and ATG5 targeted by miR-30b. In contrast to miR-30b overexpression, its inhibition ultimately causes the upregulation of autophagy and represses apoptosis and cartilage degradation. Overall, miR-30b can be regarded as a key factor in maintaining the balance between autophagy and apoptosis induced by TNF-*α*. Song et al. [71] subsequently performed an additional study and clarified that suppression of chondrocyte autophagy, which was related to decreased expression of Beclin1, strongly correlated with progression of OA via PI3K/AKT/mTOR. That suggests that there might be a potential signaling pathway initiated by miR-30b, the specific mechanism of which remains undiscovered in OA cartilage.

### 3.4. miR-146a

The function of miR-146a in OA remains unclear. Li et al. [72] discovered the induction of miR-146a in chondrocyte apoptosis and a few years later, a further research study that simulated the mechanical pressure of OA on chondrocytes* in vitro*, found targeting effects towards Smad4 by miR-146a during mechanical injury [11]. Nonetheless, Smad4 was considered not to be connected with chondrocyte autophagy, while the role of the novel target genes TNF receptor associated factor 6 (TRAF6) and IL-1 receptor associated kinase 1 (IRAK1) was established in the meantime [49]. TRAF6 is a ubiquitin ligase that performs a role in cell signal transmission. Activated TRAF6 can bind to phosphorylated IRAK1 and initiate NF-*κ*B signaling, an important process that mediates inflammatory cytokines and reactions during degradation of OA cartilage [73]. According to the basic experimental results of chondrocyte autophagy induced by miR-146a via Bcl-2 and the mismatch between miR-146a and Bcl-2 mRNA [50], intense research established that TRAF6 and IRAK1 are direct targets of miR-146a and intermediate factors of the regulation of Bcl-2 in hypoxia-induced chondrocyte autophagy [49]. Considering these results, the induction of hypoxia inducible factor-1*α* (HIF-1*α*) should be highlighted. HIF-1*α* can be detected in both OA and normal cartilage [74], the expression levels of which may be influenced by inflammatory cytokines or growth factors [75]. Beclin1/Bcl-2 modulation [76], AMPK activation, and mTOR suppression [77] are involved in autophagy induced by HIF-1*α*. During hypoxia-related processes, no increase in miR-146a has been observed after the blocking of HIF-1*α* [50]. In general, HIF-1*α* acts as an upstream site and directly targets miR-146a, regulating downstream genes and inducing autophagy in chondrocytes.

### 3.5. miR-17-5p

miR-17-5p, a member of the miR-17~92 cluster, is vital for growth and skeletal development [78]. miR-17-5p has received more attention in the field of autophagy in recent years. Sequestosome-1/p62 (SQSTM1), known as a selective autophagy adaptor protein, plays an essential role in ubiquitin-mediated protein degradation. The decreased expression of p62 has already been reported on account of the ubiquitination pathway in the process of autophagy [79]. Recent research has demonstrated that autophagy is promoted by miR-17-5p through p62 in SW1353 human chondrosarcoma cells [51]. Overexpression of miR-17-5p suppresses p62 expression and increases LC3 dots (punctate spots) in cell experiments, consequently activating autophagy, with researchers also obtaining the same results in experimental OA animal models. Decreased LC3 puncta and increased P62 protein levels in knee joints of OA mice have been observed [51]. According to these findings, it is speculated that there may be similarly low levels of miR-17-5p and autophagy in human OA cartilage. In other non-OA studies, several ATGs were found to be associated with miR-17-5p, such as ULK1 [52], Beclin1 [53], and myeloid cell leukemia-1 (Mcl-1, an antiapoptotic Bcl-2 family member) [54]. But whether the autophagic mechanisms of these genes exist in cartilage still require investigation.

### 3.6. miR-21

In spite of definite inhibition of autophagy by miR-21 being established in nucleus pulposus cells [55], little progress has been achieved in chondrocytes. Early miRNA microarray research discovered differential expression of miR-21 in cartilage [80]. Song et al. [56] demonstrated reduced expression of miR-21 in OA compared to normal cells and the downregulation of ATGs and LC3B induced by the suppression of miR-21. Furthermore, they also ascertained the critical role of growth arrest-specific transcript 5 (GAS5) in chondrocyte autophagy through suppression of miR-21 for the first time. GAS5, belonging to the class of long noncoding RNAs (lncRNAs), serves as a sponge to bind to a series of miRNAs, blocking the interaction between miRNAs and mRNAs. The induction of exogenous GAS5 is able to reduce the expression of miR-21 and lead to the downregulation of Beclin1, ATG7, and LC3B, indicating a decreased level of autophagy [56]. Another researcher predicted that growth differentiation factor 5 (GDF5) was a potential downstream target of miR-21 in chondrocytes, finally proving that it was [57]. Nevertheless, there is no definite evidence to verify the targeting association between them in autophagy. Findings of higher miR-21 expression in OA from Zhang et al. [57] contradict the research of Song et al. [56]. Different tissue sources and stage of OA contribute to this divergence. The former research compared cartilage specimens in OA with that in traumatic amputees, while the latter compared the femoral condyle and tibial plateau, using a relatively normal concept of OA. Prior to clinical patients receiving a total knee replacement, the stages of OA disease are not static. That is to say, not only does the degree of cartilage degeneration interfere with miR-21 expression and autophagy, but so too do other factors, such as mechanical pressure and inflammation.

### 3.7. Emerging miRNAs

In addition to the miRNAs mentioned above, which have been investigated in depth, a few miRNAs have just come to the fore. miR-335-5p has been found in OA cartilage in which it directly promoted autophagy of chondrocytes [58]. However, the specific mechanisms or pathways of miR-335-5p were not elucidated. Several miRNAs, including miR-9 [59] and miR-449a [60], have been considered to directly target SIRT1, which has protective effects and regulate autophagy in chondrocytes. In recent research, miR-4262 was also demonstrated to be effective in these miRNAs. miR-4262 overexpression resulted in the inhibition of SIRT1 and activation of PI3K/AKT/mTOR, ultimately decreasing chondrocyte autophagy and inducing the development of OA [61]. The PI3K/AKT/mTOR signaling pathway has been mentioned above due to its vital role in autophagy. This pathway encompasses a series of associated genes, proteins, or cytokines, explaining why miRNAs have effects on autophagy by targeting these factors. For instance, researchers have recently clarified the negative regulation of miR-206 targeting insulin-like growth factor-1 (IGF-1) in autophagy through this pathway [62]. Another notable miRNA is miR-20 [63], whose mechanisms in the PI3K/AKT/mTOR pathway are similar to those of miR-206. However, inhibition of autophagy is dependent on the targeting effect of miR-20 on ATG10 too. ATG10, along with ATG12 which is targeted by miR-128a [64], is essential in the elongation process. Silencing of these ATGs triggers the termination of autophagy. Zhao et al. [65] identified miR-107 as a promoter of autophagy in OA models. They also discovered its target TRAF3 and inhibition of AKT/mTOR activation under miR-107 overexpression. Besides, miRNAs within OA pathophysiological factors have also been discussed. It was reported that proliferation, apoptosis, and autophagy in chondrocytes could be influenced by miR-140-5p and miR-149 [66]. Fucosyltransferase 1 (FUT1) was the direct target, and disorders of glycosylated protein modification mediated by FUT1 are responsible for aberrant autophagy. Wang et al. [67] simulated the pathogenesis of OA by exerting mechanical pressure on primary chondrocytes. They found that increased expression of miR-590-5p in experimental models demonstrated promotion of chondrocyte autophagy via transforming growth factor *β*1 (TGF-*β*1). Similar to miR-146a, miR-590-5p is also involved in pressure-mediated cartilage degeneration.

### 3.8. Potentially Relevant miRNAs

Although autophagy has been widely studied, the mechanisms of the autophagic network of miRNAs in chondrocytes still require further investigation and to be better understood. Apart from their identification, there are indications that a number of other miRNAs are potential candidates, whose differential expressions in OA cartilage have already been elucidated. Furthermore, through noncartilage research it has been established that they are also involved in autophagic processes in other tissues. A list of these miRNAs is presented in Table 2.

miR-140 is the most promising owing to its high cartilage specificity in numerous basic research studies [131]. Wang et al. [66] demonstrated a targeting relationship between miR-140-5p and FUT1. Meanwhile ULK1 [81], a novel target of miR-140 (miR-140-5p), has also been identified in 293T cells. It can be predicted that the same mechanisms might exist in OA chondrocytes, and intervention of miR-140 in autophagy remains to be further clarified. miR-93 is an additional miRNA that was found to target ULK1, mediating hypoxia-induced autophagy in either MEFs or CHO cells [83]. Similar to miR-93, miR-26a-5p shares the same target [86]. Thus, one can speculate that it is a possibility that miR-93 and miR-26a-5p participate in regulating the balance of autophagy in chondrocytes. According to the analyses of Haseeb et al. [85] and Akhtar et al. [113], miR-27b-3p is considered to be the most abundant miRNA in OA cartilage, possibly regulating the expression of MMP13 in chondrocytes. In consideration of the autophagic clearance of mitochondria induced by miR-27b-3p [114], miR-27b-3p is likely to be involved in a number of undiscovered autophagic activities in chondrocytes. Recently, miR-22-3p has been investigated for MTDH-mediated autophagy in the regulation of proliferation and sensitivity in osteosarcoma cells [122]. With high expression levels of miR-22-3p in cartilage [91], researchers will show much interest in its induction and pathways of chondrocyte autophagy. Research in OA cartilage has confirmed differential expression of miRNAs, including miR-218-5p, miR-634, miR-145, and miR-30a-5p [47, 97–99]. Since PI3K/AKT/mTOR represents a common pathway used by miR-218-5p, miR-634, and miR-145, degrees of autophagy can be regulated due to potential targeted upstream or downstream factors. Interestingly, the findings also suggest that miR-30a-5p targets AKT genes during the apoptosis of chondrocytes. Other teams have ascertained several additional miRNAs which modulate mTOR signaling, including miR-302b, mir-148a-3p, miR-99a-5p, miR-222-3p, and miR-199a-3p [104, 105, 107, 109, 112]. Taken together, as mTOR is at the core of autophagy, any interventions are likely to regulate the process in chondrocytes. Furthermore, regarded as a target towards SIRT1, miR-34a behaves in accordance with miR-449a [28, 60]. Other target genes of miR-34a, including ATG4B and ATG9A, were also identified. Considering the upstream circRNA.2837, which acts as a sponge to miR-34a, its knockdown could induce neuronal autophagy* in vivo* [119–121]. It is particularly important to understand whether miR-34a modulates autophagy in OA cartilage.

## 4. Conclusions and Future Perspectives

Failure of homeostasis modulated by miRNAs in chondrocyte autophagy represents a pivotal mechanism in the progression of OA. Although achievements have been obtained, understanding of miRNA interventions in autophagy is still in its infancy. Under the constant identification of ATGs, additional miRNAs that have a potential role will be explored in the near future. A giant regulatory network of autophagy among miRNAs, target genes, and signaling pathways is gradually emerging as bioinformatic prediction has increased in popularity. Independent one-to-many or many-to-one targeting relationships and specific crosstalk effects are crucial in interfering with chondrocyte autophagy, which remains indefinite and has become a great challenge. Moreover, the theory of competing endogenous RNAs (ceRNAs) reveals the existence of an upstream regulatory pathway referring to lncRNAs or circular RNAs (circRNAs), breaking new ground in miRNA research. Referring to recent studies, lncRNA-ROR (lncRNA-regulator of reprogramming, involved in chondrocyte proliferation and apoptosis) and lncRNA-CIR (OA cartilage injury-related lncRNA) have been demonstrated to directly participate in OA progression by modulation of autophagy [132, 133]. Compared with these findings, there are no explicit or similar reports about circRNAs in OA chondrocytes. In other words, research on circRNAs and their target miRNAs are urgently required to achieve a more complete understanding of chondrocyte autophagy.

Gene interference by miRNAs has become a promising and required direction in the therapy of maintenance of autophagy. Interventions in several known miRNAs have been conducted in both* in vitro* and* in vivo* models, such as miR-206 inhibitor and miR-128a antisense oligonucleotide [62, 64], ultimately achieving satisfactory autophagy recovery and chondrocyte survival. However, autophagy is not the sole factor that determines the fate of chondrocytes. OA progression is holistic and closely associated with chondrocyte autophagy, apoptosis, and senescence, in which miRNAs are likely to participate simultaneously. Considering the multitargeting character of miRNAs, single functional studies appear insufficient to elucidate the complex autophagic network. Comprehensive research on chondrocyte function regulated by miRNAs is required before clinical application of gene manipulation in autophagy is utilized.

## Figures and Tables

**Table 1:** miRNAs and their targets that have been identified in the autophagy of OA chondrocytes.

microRNAs	Targets (upstream or downstream)	Mechanisms	Research cells or models	References
miR-155	ULK1, FOXO3, ATG3, ATG5, ATG14, GABARAPL1 and MAP1LC3	Autophagy	Human chondrocytes, T/C28a2 cells	[43]

miR-155	Rictor	mTORC2 signaling	MCF-7, MDA-MB-157, BT-549 human breast cancer cell lines	[44]

miR-30b	Beclin1, ATG5	Autophagy and apoptosis	ATDC5 cells	[45]

miR-30a, miR-30b, miR-30c, miR-30d, miR-30e	Beclin1	Autophagy	Adipocytes	[46]

miR-30a-5p	AKT	Cell cycle and apoptosis	Human osteoarthritic chondrocytes, SW1353 cells	[47]

miR-30c	ATG5, ATG16L1	Autophagy	Human intestinal epithelial T84 cells	[48]

miR-146a	TRAF6, IRAK1	Autophagy	C57BL/6J mouse chondrocytes	[49]

miR-146a	Bcl2	Autophagy	C57BL/6J mouse chondrocytes	[50]

miR-146a	HIF-1*α*	Autophagy	C57BL/6J mouse chondrocytes	[50]

miR-17-5p	SQSTM1/p62	Autophagy	C57BL/6J mouse, SW1353 human chondrosarcoma cells	[51]

miR-17-5p	ULK1	Growth modulation	Murine macrophage RAW264.7 cells, human HEK 293T cells	[52]

miR-17-5p	Beclin1	Autophagy-related resistance	A549 and H596 lung cancer cells	[53]

miR-17-5p	Mcl-1, STAT3	Autophagy	Murine macrophage cell line RAW264.7, HEK293 cells	[54]

miR-21	PTEN/AKT/mTOR	Autophagy	Human degenerated nucleus pulposus cells	[55]

miR-21	GAS5	Autophagy	Human osteoarthritic chondrocytes	[56]

miR-21	GDF5	Chondrogenesis	Human articular chondrocytes, CH8 cell lines	[57]

miR-335-5p	Unknown	Autophagy, proliferation and apoptosis	Human osteoarthritic chondrocytes	[58]

miR-9	SIRT1	Autophagy and protection	Human chondrocytes, C-28/I2 cells	[59]

miR-449a	SIRT1	Autophagy and protection	Human chondrocytes, SW1353 cells	[60]

miR-4262	SIRT1, PI3K/AKT/mTOR	Autophagy	SD rat chondrocytes	[61]

miR-206	IGF-1, PI3K/AKT/mTOR	Autophagy and apoptosis	Wistar rat chondrocytes	[62]

miR-20	ATG10, PI3K/AKT/mTOR	Autophagy and proliferation	SD rat chondrocytes	[63]

miR-128a	ATG12	Autophagy	Human chondrocytes, SD rat chondrocytes	[64]

miR-107	TRAF3, AKT/mTOR	Autophagy and apoptosis	Human osteoarthritic chondrocytes, SD rats	[65]

miR-140-5p, miR-149	FUT1	Autophagy, apoptosis and proliferation	Human osteoarthritic chondrocytes	[66]

miR-590-5p	TGF-*β*1	Autophagy and apoptosis	Human chondrocytes	[67]

**Table 2:** Predicted miRNAs and their potential targets in autophagy of OA chondrocytes.

microRNAs	Differential expression in OA cartilage, References	Potential related targets or genes	Mechanisms or functions	Research diseases, cells or models	References
miR-140-5p	[66]	ULK1	Autophagy	Human HEK 293T cells	[81]

miR-93	[82]	ULK1	Autophagy	Mouse embryonic fibroblasts (MEFs), Chinese hamster ovary cells (CHO)	[83]

miR-26a-5p	[84, 85]	ULK1	Autophagy	Primary cardiac fibroblasts from neonatal rats	[86]

miR-25	[87]	ULK1	Autophagy	Human breast cancer cells (MCF-7), normal human mammary epithelial cell (MCF-10A)	[88]

miR-558	[12, 89]	lncRNA MALAT1, ULK1	Autophagy and apoptosis	Rat myocardial cells, H9C2	[90]

miR-210	[91]	ATG7	Autophagy	Human lumbar degenerated NP cells	[92]

miR-29a	[1, 91]	TFEB, ATG9A	Autophagy	Human pancreatic epithelial cells, HPNE and HPDE	[93]

miR-23b-3p	[87]	ATG12	Autophagy	Traumatic brain injury, SD rats	[94]

miR-125b-5p	[85, 95]	UVRAG	Autophagy	Systemic lupus erythematosus, human PBMCs	[96]

miR-218-5p	[97]	PIK3C2A, PI3K/AKT/mTOR	Matrix synthesis, proliferation and apoptosis	Human osteoarthritic chondrocytes, SW1353 and C28/I2 cells	[97]

miR-634	[98]	PIK3R1, PI3K/AKT/mTOR/S6	Matrix synthesis and survival	Human osteoarthritic chondrocytes, HEK293 cells	[98]

miR-145	[95, 99, 100]	PI3K/AKT/mTOR	Autophagy	Human umbilical cord-derived mesenchymal stem cells, HK-2 cell	[101]

miR-181b	[102]	PTEN/Akt/mTOR	Autophagy	Parkinson's disease, PC12 cells	[103]

miR-302b	[104]	Smad3, Notch2, mTOR pathway	Inflammation suppression	Human chondrocytes, C-28/I2 cells	[104]

mir-148a-3p	[85, 95]	RAB12, mTOR1	Autophagy	Gastric cancer, BGC823/CDDP and SGC7901/CDDP cells	[105]

miR-99a-5p	[106]	mTOR	Autophagy and apoptosis	Human immortalized uroepithelial cells (SV-Huc1), bladder cancer cells 5637 (HTB-9) and T24 (HTB-4)	[107]

miR-222-3p	[108]	ATG12/p27-mTOR	Autophagy	Human MM cells, MM.1S, MM.1R, RPMI-8226, U266, NCIH929, and ARH-77	[109]

miR-199a-3p	[110, 111]	IGF-1, mTOR	Autophagy	Osteocyte-like MLO-Y4 cells	[112]

miR-27b-3p	[85, 113]	PTEN-induced putative kinase 1 (PINK1)	Autophagy	Human cervical HeLa, dopaminergic-like M17 cells	[114]

miR-24	[115]	SIRT1, deacetylated LC3	Autophagy	Uterine sarcoma	[116]

miR-138-5p	[117]	SIRT1	Autophagy	Human neuroblastoma cells (SH-SY5Y)	[118]

miR-34a	[28]	ATG4B	Autophagy	Tubular epithelial cells	[119]

miR-34a	[28]	ATG9A	Autophagy	SD rat cardiomyocytes	[120]

miR-34a	[28]	circRNA.2837	Neuronal autophagy	SD rat spinal neurons	[121]

miR-22-3p	[85, 91]	Metadherin (MTDH)	Autophagy	Human osteosarcoma cells (MG-63)	[122]

miR-377	[91, 100]	Rapamycin	Autophagy	Murine macrophage RAW264.7 cells	[123]

miR-103	[91, 100]	SOX2	Autophagy and apoptosis	LPS-injured PC12 cells, SD rats	[124]

miR-193b	[110]	Stathmin 1	Autophagy and non-apoptotic cell death	Human oesophageal cancer cells, OE19, OE21 and OE33	[125]

miR-16-5p	[87]	Guanine nucleotide-binding *α*-subunit12 (G*α*12)	Autophagy	Hepatic stellate cells, HSCs	[126]

miR-320	[95, 127]	HIF-1*α*	Autophagy	Human RB cells (WERI-RB1)	[128]

miR-195	[129]	GABARAPL1	Autophagy, proliferation, migration and angiogenesis	Human endothelial progenitor cells (hEPCs)	[130]
